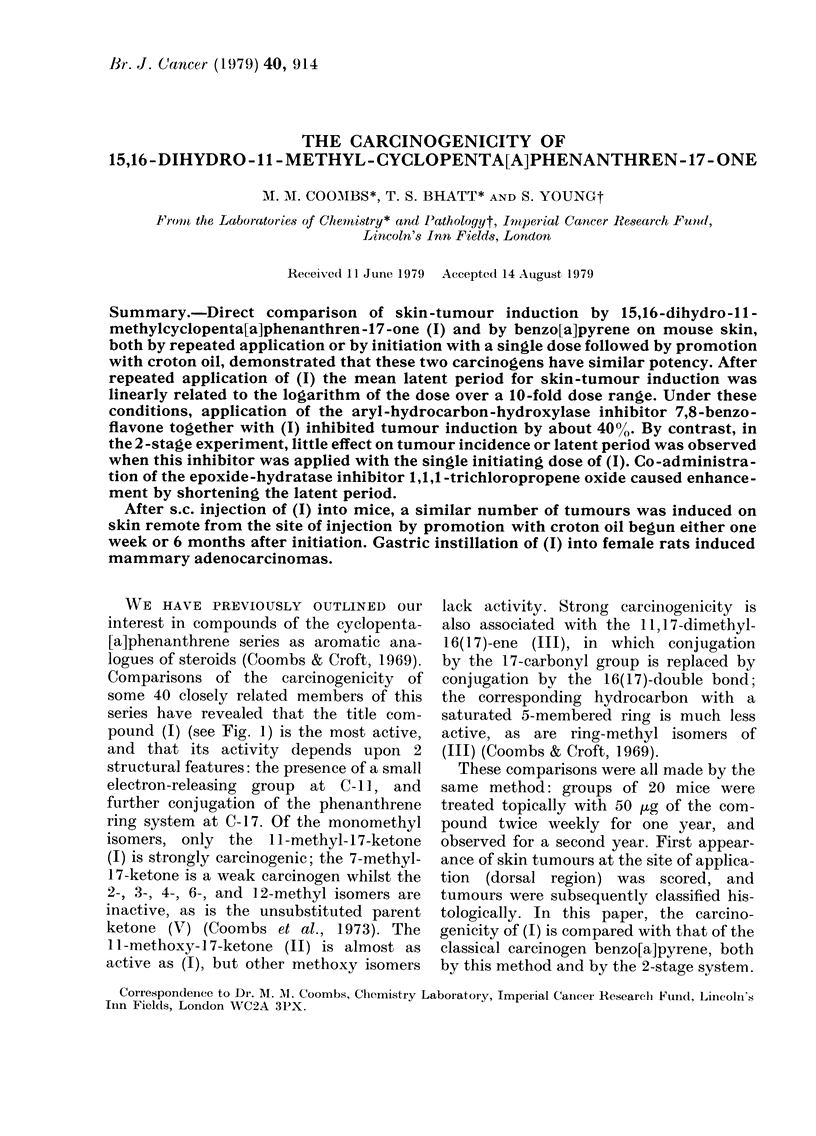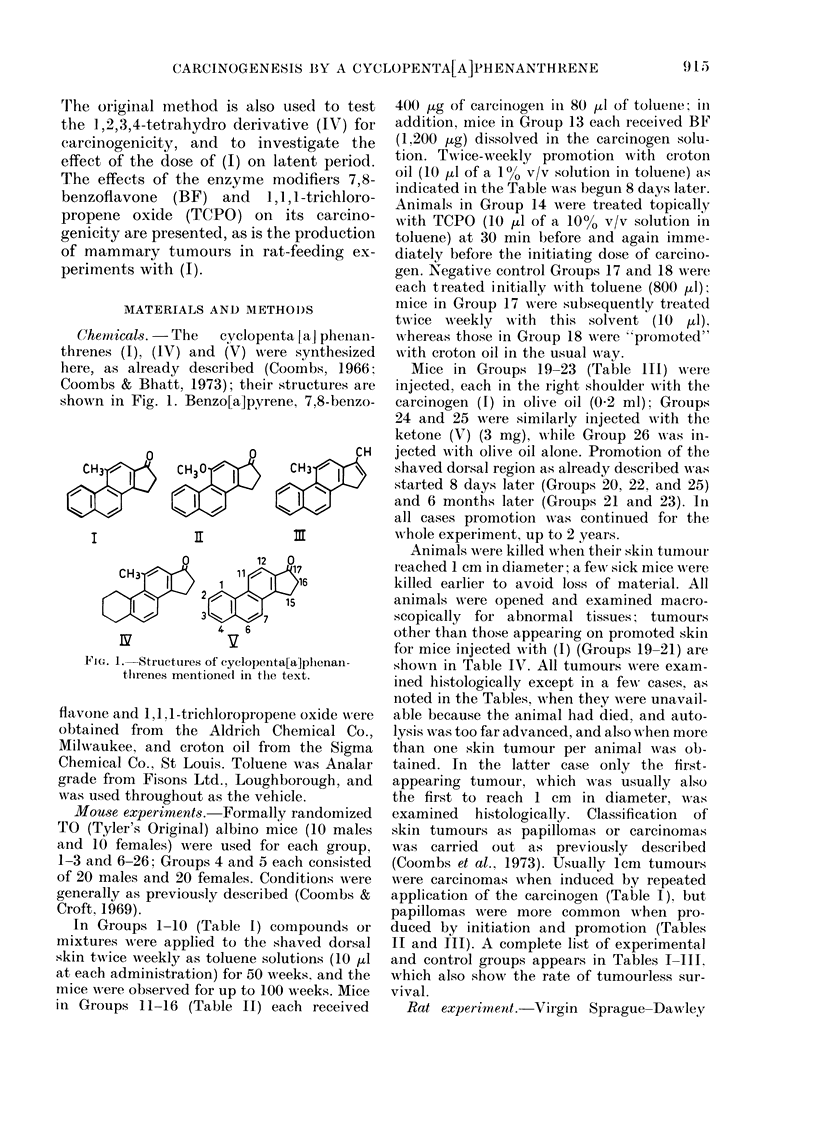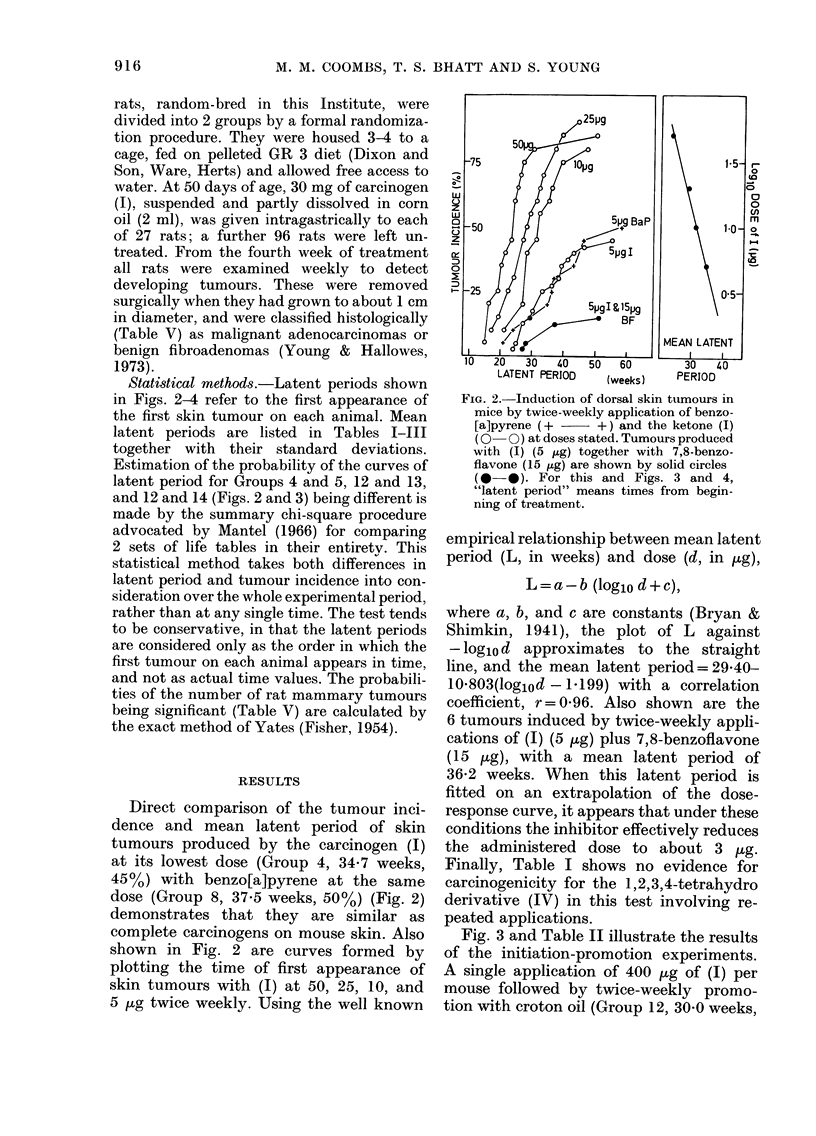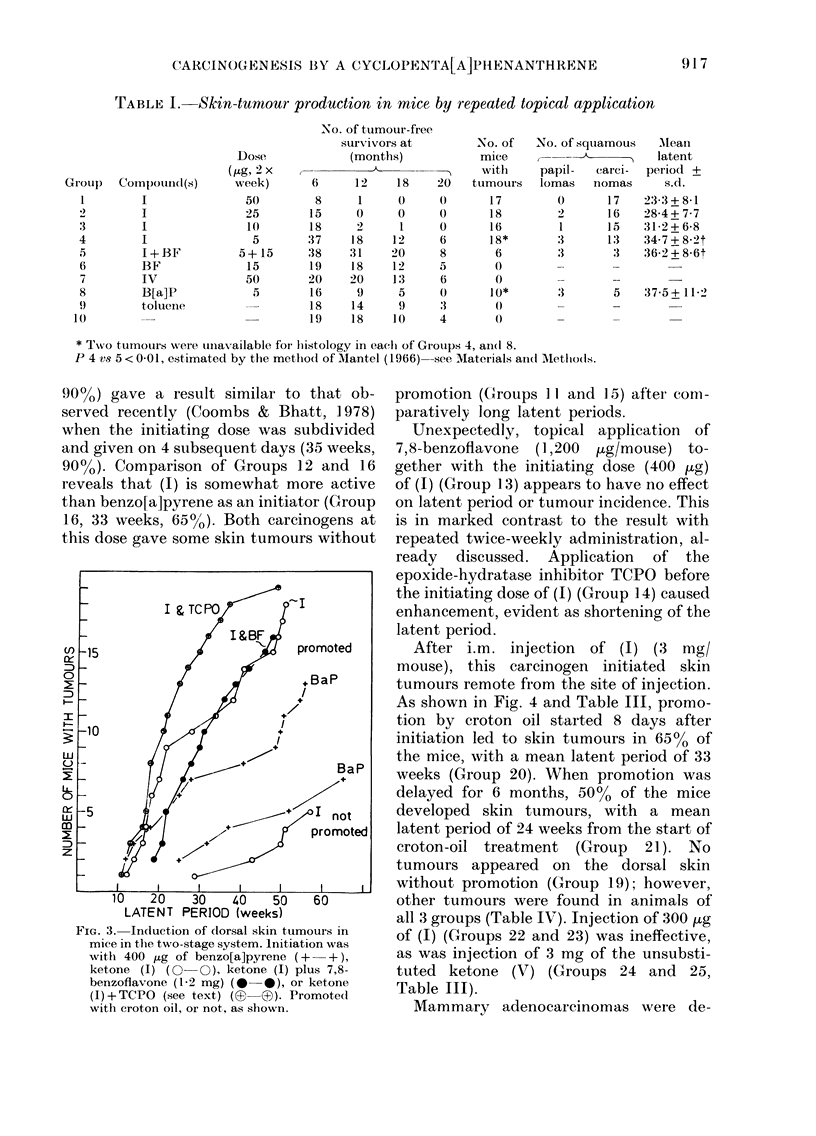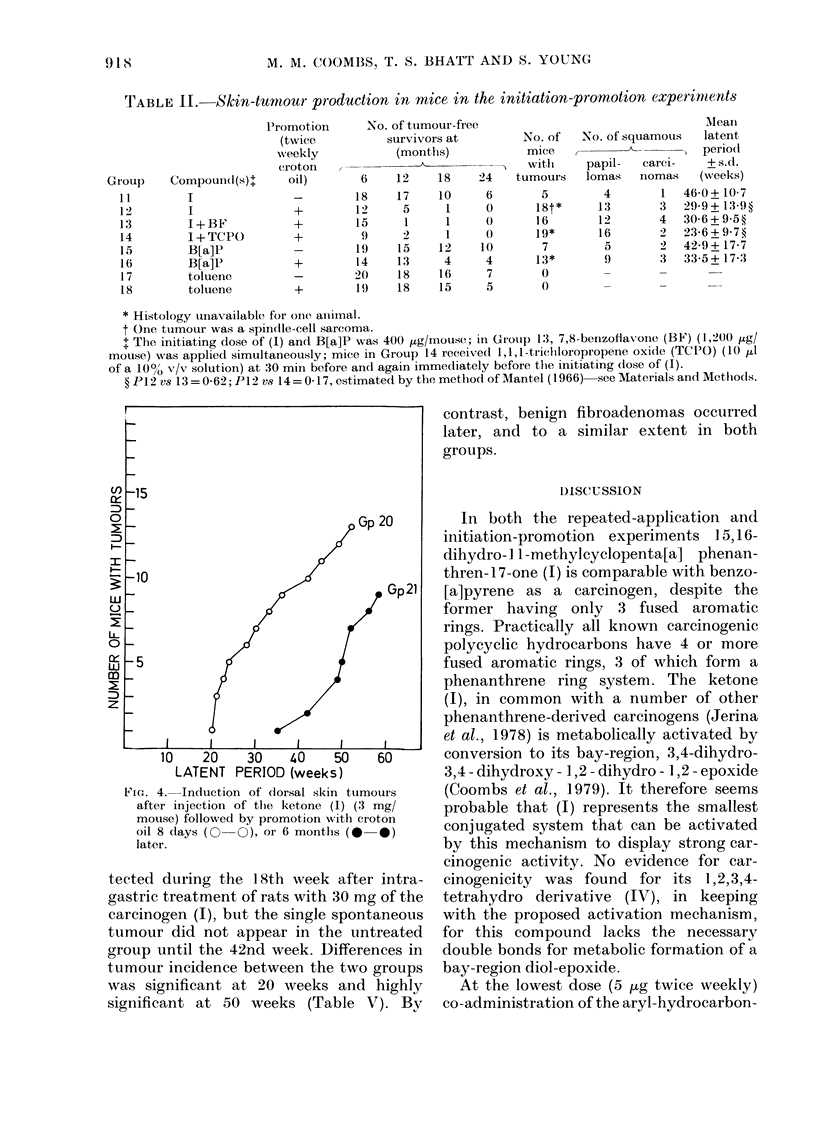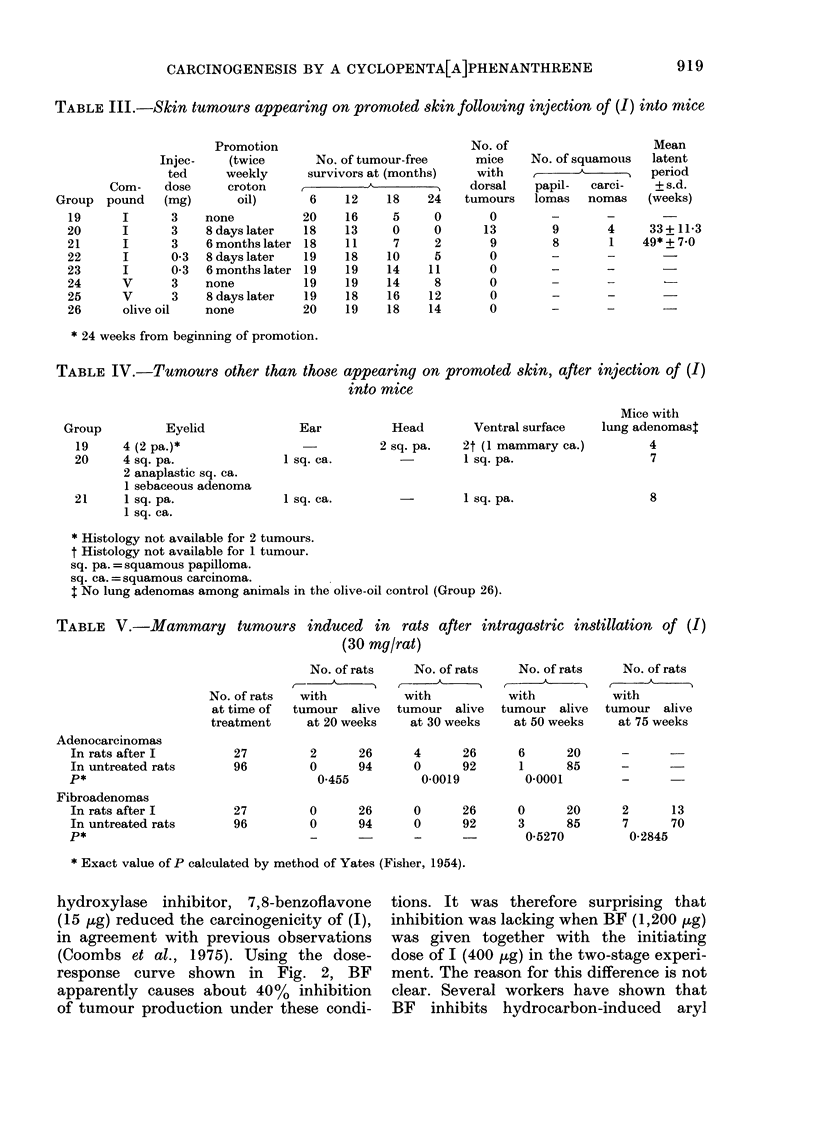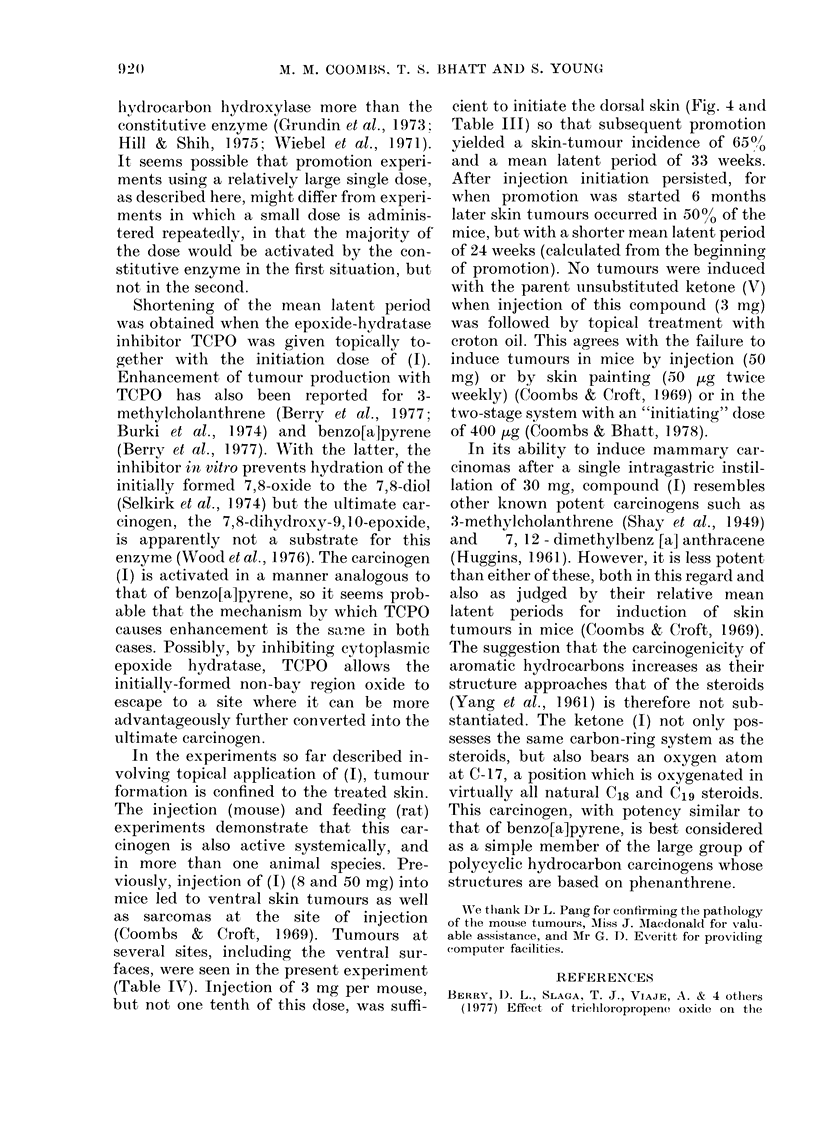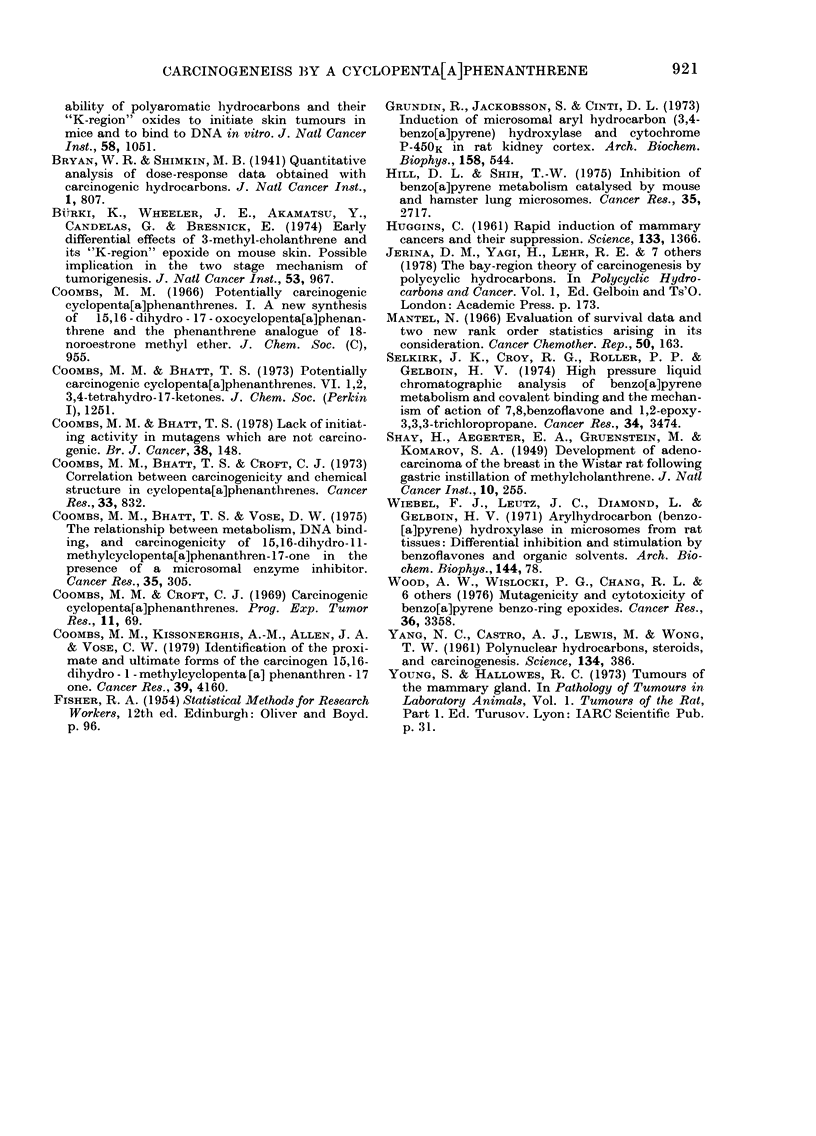# The carcinogenicity of 15,16-dihydro-11-methyl-cyclopenta[a]phenanthren-17-one.

**DOI:** 10.1038/bjc.1979.285

**Published:** 1979-12

**Authors:** M. M. Coombs, T. S. Bhatt, S. Young

## Abstract

Direct comparison of skin-tumour induction by 15,16-dihydro-11-methylcyclopenta[a]phenanthren-17-one (I) and by benzo[a]pyrene on mouse skin, both by repeated application or by initiation with a single dose followed by promotion with croton oil, demonstrated that these two carcinogens have similar potency. After repeated application of (I) the mean latent period for skin-tumour induction was linearly related to the logarithm of the dose over a 10-fold dose range. Under these conditions, application of the aryl-hydrocarbon-hydroxylase inhibitor 7,8-benzoflavone together with (I) inhibited tumour induction by about 40%. By contrast, in the 2-stage experiment, little effect on tumour incidence or latent period was observed when this inhibitor was applied with the single initiating dose of (I). Co-administration of the epoxide-hydratase inhibitor 1,1,1-trichloropropene oxide caused enhancement by shortening the latent period. After s.c. injection of (I) into mice, a similar number of tumours was induced on skin remote from the site of injection by promotion with corton oil begun either one week or 6 months after initiation. Gastric instillation of (I) into female rats induced mammary adenocarcinomas.


*Br. J. Cancer* (1979) **40**, 914

### THE CARCINOGENICITY OF 15,16-DIHYDRO-11-METHYL-CYCLOPENTA[A]PHENANTHREN-17-ONE

M. M. COOMBS*, T. S. BHATT* AND S. YOUNG†

*From the Laboratories of Chemistry* and Pathology†, Imperial Cancer Research Fund, Lincoln's Inn Fields, London*

Received 11 June 1979 Accepted 14 August 1979

**Summary.—Direct comparison of skin-tumour induction by 15,16-dihydro-11-methylcyclopenta[a]phenanthren-17-one (I) and by benzo[a]pyrene on mouse skin, both by repeated application or by initiation with a single dose followed by promotion with croton oil, demonstrated that these two carcinogens have similar potency. After repeated application of (I) the mean latent period for skin-tumour induction was linearly related to the logarithm of the dose over a 10-fold dose range. Under these conditions, application of the aryl-hydrocarbon-hydroxylase inhibitor 7,8-benzoflavone together with (I) inhibited tumour induction by about 40%. By contrast, in the 2-stage experiment, little effect on tumour incidence or latent period was observed when this inhibitor was applied with the single initiating dose of (I). Co-administration of the epoxide-hydratase inhibitor 1,1,1-trichloropropene oxide caused enhancement by shortening the latent period.**

**After s.c. injection of (I) into mice, a similar number of tumours was induced on skin remote from the site of injection by promotion with croton oil begun either one week or 6 months after initiation. Gastric instillation of (I) into female rats induced mammary adenocarcinomas.**

WE HAVE PREVIOUSLY OUTLINED our interest in compounds of the cyclopenta[a]phenanthrene series as aromatic analogues of steroids (Coombs & Croft, 1969). Comparisons of the carcinogenicity of some 40 closely related members of this series have revealed that the title compound (I) (see Fig. 1) is the most active, and that its activity depends upon 2 structural features: the presence of a small electron-releasing group at C-11, and further conjugation of the phenanthrene ring system at C-17. Of the monomethyl isomers, only the 11-methyl-17-ketone (I) is strongly carcinogenic; the 7-methyl-17-ketone is a weak carcinogen whilst the 2-, 3-, 4-, 6-, and 12-methyl isomers are inactive, as is the unsubstituted parent ketone (V) (Coombs *et al.*, 1973). The 11-methoxy-17-ketone (II) is almost as active as (I), but other methoxy isomers lack activity. Strong carcinogenicity is also associated with the 11,17-dimethyl-16(17)-ene (III), in which conjugation by the 17-carbonyl group is replaced by conjugation by the 16(17)-double bond; the corresponding hydrocarbon with a saturated 5-membered ring is much less active, as are ring-methyl isomers of (III) (Coombs & Croft, 1969).

These comparisons were all made by the same method: groups of 20 mice were treated topically with 50 μg of the compound twice weekly for one year, and observed for a second year. First appearance of skin tumours at the site of application (dorsal region) was scored, and tumours were subsequently classified histologically. In this paper, the carcinogenicity of (I) is compared with that of the classical carcinogen benzo[a]pyrene, both by this method and by the 2-stage system.

Correspondence to Dr. M. M. Coombs, Chemistry Laboratory, Imperial Cancer Research Fund, Lincoln's Inn Fields, London WC2A 3PX.

The original method is also used to test the 1,2,3,4-tetrahydro derivative (IV) for carcinogenicity, and to investigate the effect of the dose of (I) on latent period. The effects of the enzyme modifiers 7,8-benzoflavone (BF) and 1,1,1-trichloropropene oxide (TCPO) on its carcinogenicity are presented, as is the production of mammary tumours in rat-feeding experiments with (I).

### MATERIALS AND METHODS

*Chemicals.*—The cyclopenta[a]phenanthrenes (I), (IV) and (V) were synthesized here, as already described (Coombs, 1966; Coombs & Bhatt, 1973); their structures are shown in Fig. 1. Benzo[a]pyrene, 7,8-benzo-

FIG. 1.—Structures of cyclopenta[a]phenanthrenes mentioned in the text.

flavone and 1,1,1-trichloropropene oxide were obtained from the Aldrich Chemical Co., Milwaukee, and croton oil from the Sigma Chemical Co., St Louis. Toluene was Analar grade from Fisons Ltd., Loughborough, and was used throughout as the vehicle.

*Mouse experiments.*—Formally randomized TO (Tyler's Original) albino mice (10 males and 10 females) were used for each group, 1–3 and 6–26; Groups 4 and 5 each consisted of 20 males and 20 females. Conditions were generally as previously described (Coombs & Croft, 1969).

In Groups 1–10 (Table I) compounds or mixtures were applied to the shaved dorsal skin twice weekly as toluene solutions (10 μl at each administration) for 50 weeks, and the mice were observed for up to 100 weeks. Mice in Groups 11–16 (Table II) each received 400 μg of carcinogen in 80 μl of toluene; in addition, mice in Group 13 each received BF (1,200 μg) dissolved in the carcinogen solution. Twice-weekly promotion with croton oil (10 μl of a 1% v/v solution in toluene) as indicated in the Table was begun 8 days later. Animals in Group 14 were treated topically with TCPO (10 μl of a 10% v/v solution in toluene) at 30 min before and again immediately before the initiating dose of carcinogen. Negative control Groups 17 and 18 were each treated initially with toluene (800 μl); mice in Group 17 were subsequently treated twice weekly with this solvent (10 μl), whereas those in Group 18 were "promoted" with croton oil in the usual way.

Mice in Groups 19–23 (Table III) were injected, each in the right shoulder with the carcinogen (I) in olive oil (0·2 ml); Groups 24 and 25 were similarly injected with the ketone (V) (3 mg), while Group 26 was injected with olive oil alone. Promotion of the shaved dorsal region as already described was started 8 days later (Groups 20, 22, and 25) and 6 months later (Groups 21 and 23). In all cases promotion was continued for the whole experiment, up to 2 years.

Animals were killed when their skin tumour reached 1 cm in diameter; a few sick mice were killed earlier to avoid loss of material. All animals were opened and examined macroscopically for abnormal tissues; tumours other than those appearing on promoted skin for mice injected with (I) (Groups 19–21) are shown in Table IV. All tumours were examined histologically except in a few cases, as noted in the Tables, when they were unavailable because the animal had died, and autolysis was too far advanced, and also when more than one skin tumour per animal was obtained. In the latter case only the first-appearing tumour, which was usually also the first to reach 1 cm in diameter, was examined histologically. Classification of skin tumours as papillomas or carcinomas was carried out as previously described (Coombs *et al.*, 1973). Usually 1cm tumours were carcinomas when induced by repeated application of the carcinogen (Table I), but papillomas were more common when produced by initiation and promotion (Tables II and III). A complete list of experimental and control groups appears in Tables I–III, which also show the rate of tumourless survival.

*Rat experiment.*—Virgin Sprague–Dawley rats, random-bred in this Institute, were divided into 2 groups by a formal randomization procedure. They were housed 3–4 to a cage, fed on pelleted GR 3 diet (Dixon and Son, Ware, Herts) and allowed free access to water. At 50 days of age, 30 mg of carcinogen (I), suspended and partly dissolved in corn oil (2 ml), was given intragastrically to each of 27 rats; a further 96 rats were left untreated. From the fourth week of treatment all rats were examined weekly to detect developing tumours. These were removed surgically when they had grown to about 1 cm in diameter, and were classified histologically (Table V) as malignant adenocarcinomas or benign fibroadenomas (Young & Hallowes, 1973).

*Statistical methods.*—Latent periods shown in Figs. 2–4 refer to the first appearance of the first skin tumour on each animal. Mean latent periods are listed in Tables I–III together with their standard deviations. Estimation of the probability of the curves of latent period for Groups 4 and 5, 12 and 13, and 12 and 14 (Figs. 2 and 3) being different is made by the summary chi-square procedure advocated by Mantel (1966) for comparing 2 sets of life tables in their entirety. This statistical method takes both differences in latent period and tumour incidence into consideration over the whole experimental period, rather than at any single time. The test tends to be conservative, in that the latent periods are considered only as the order in which the first tumour on each animal appears in time, and not as actual time values. The probabilities of the number of rat mammary tumours being significant (Table V) are calculated by the exact method of Yates (Fisher, 1954).

### RESULTS

Direct comparison of the tumour incidence and mean latent period of skin tumours produced by the carcinogen (I) at its lowest dose (Group 4, 34·7 weeks, 45%) with benzo[a]pyrene at the same dose (Group 8, 37·5 weeks, 50%) (Fig. 2) demonstrates that they are similar as complete carcinogens on mouse skin. Also shown in Fig. 2 are curves formed by plotting the time of first appearance of skin tumours with (I) at 50, 25, 10, and 5 μg twice weekly. Using the well known

FIG. 2.—Induction of dorsal skin tumours in mice by twice-weekly application of benzo[a]pyrene (+ —— +) and the ketone (I) (○—○) at doses stated. Tumours produced with (I) (5 μg) together with 7,8-benzoflavone (15 μg) are shown by solid circles (●—●). For this and Figs. 3 and 4, "latent period" means times from beginning of treatment.

empirical relationship between mean latent period (L, in weeks) and dose ($d$, in μg),

$$L = a - b\ (\log_{10} d + c),$$

where $a$, $b$, and $c$ are constants (Bryan & Shimkin, 1941), the plot of L against $-\log_{10} d$ approximates to the straight line, and the mean latent period = 29·40–10·803($\log_{10} d$ − 1·199) with a correlation coefficient, $r = 0{\cdot}96$. Also shown are the 6 tumours induced by twice-weekly applications of (I) (5 μg) plus 7,8-benzoflavone (15 μg), with a mean latent period of 36·2 weeks. When this latent period is fitted on an extrapolation of the dose-response curve, it appears that under these conditions the inhibitor effectively reduces the administered dose to about 3 μg. Finally, Table I shows no evidence for carcinogenicity for the 1,2,3,4-tetrahydro derivative (IV) in this test involving repeated applications.

Fig. 3 and Table II illustrate the results of the initiation-promotion experiments. A single application of 400 μg of (I) per mouse followed by twice-weekly promotion with croton oil (Group 12, 30·0 weeks,

TABLE I.—*Skin-tumour production in mice by repeated topical application*

| Group | Compound(s) | Dose ($\mu$g, 2 × week) | No. of tumour-free survivors at (months) 6 | 12 | 18 | 20 | No. of mice with tumours | No. of squamous papillomas | carcinomas | Mean latent period ± s.d. |
|---|---|---|---|---|---|---|---|---|---|---|
| 1 | I | 50 | 8 | 1 | 0 | 0 | 17 | 0 | 17 | 23·3 ± 8·1 |
| 2 | I | 25 | 15 | 0 | 0 | 0 | 18 | 2 | 16 | 28·4 ± 7·7 |
| 3 | I | 10 | 18 | 2 | 1 | 0 | 16 | 1 | 15 | 31·2 ± 6·8 |
| 4 | I | 5 | 37 | 18 | 12 | 6 | 18* | 3 | 13 | 34·7 ± 8·2† |
| 5 | I + BF | 5 + 15 | 38 | 31 | 20 | 8 | 6 | 3 | 3 | 36·2 ± 8·6† |
| 6 | BF | 15 | 19 | 18 | 12 | 5 | 0 | – | – | — |
| 7 | IV | 50 | 20 | 20 | 13 | 6 | 0 | – | – | — |
| 8 | B[a]P | 5 | 16 | 9 | 5 | 0 | 10* | 3 | 5 | 37·5 ± 11·2 |
| 9 | toluene | — | 18 | 14 | 9 | 3 | 0 | – | – | — |
| 10 | — | — | 19 | 18 | 10 | 4 | 0 | – | – | — |

* Two tumours were unavailable for histology in each of Groups 4, and 8.

† $P$ 4 *vs* 5 < 0·01, estimated by the method of Mantel (1966)—see Materials and Methods.

90%) gave a result similar to that observed recently (Coombs & Bhatt, 1978) when the initiating dose was subdivided and given on 4 subsequent days (35 weeks, 90%). Comparison of Groups 12 and 16 reveals that (I) is somewhat more active than benzo[a]pyrene as an initiator (Group 16, 33 weeks, 65%). Both carcinogens at this dose gave some skin tumours without

FIG. 3.—Induction of dorsal skin tumours in mice in the two-stage system. Initiation was with 400 $\mu$g of benzo[a]pyrene (+—+), ketone (I) (○—○), ketone (I) plus 7,8-benzoflavone (1·2 mg) (●—●), or ketone (I) + TCPO (see text) (⊕—⊕). Promoted with croton oil, or not, as shown.

promotion (Groups 11 and 15) after comparatively long latent periods.

Unexpectedly, topical application of 7,8-benzoflavone (1,200 $\mu$g/mouse) together with the initiating dose (400 $\mu$g) of (I) (Group 13) appears to have no effect on latent period or tumour incidence. This is in marked contrast to the result with repeated twice-weekly administration, already discussed. Application of the epoxide-hydratase inhibitor TCPO before the initiating dose of (I) (Group 14) caused enhancement, evident as shortening of the latent period.

After i.m. injection of (I) (3 mg/mouse), this carcinogen initiated skin tumours remote from the site of injection. As shown in Fig. 4 and Table III, promotion by croton oil started 8 days after initiation led to skin tumours in 65% of the mice, with a mean latent period of 33 weeks (Group 20). When promotion was delayed for 6 months, 50% of the mice developed skin tumours, with a mean latent period of 24 weeks from the start of croton-oil treatment (Group 21). No tumours appeared on the dorsal skin without promotion (Group 19); however, other tumours were found in animals of all 3 groups (Table IV). Injection of 300 $\mu$g of (I) (Groups 22 and 23) was ineffective, as was injection of 3 mg of the unsubstituted ketone (V) (Groups 24 and 25, Table III).

Mammary adenocarcinomas were de-

TABLE II.—*Skin-tumour production in mice in the initiation-promotion experiments*

| Group | Compound(s)‡ | Promotion (twice weekly croton oil) | No. of tumour-free survivors at (months) 6 | 12 | 18 | 24 | No. of mice with tumours | No. of squamous papil-lomas | carci-nomas | Mean latent period ± s.d. (weeks) |
|---|---|---|---|---|---|---|---|---|---|---|
| 11 | I | – | 18 | 17 | 10 | 6 | 5 | 4 | 1 | 46·0 ± 10·7 |
| 12 | I | + | 12 | 5 | 1 | 0 | 18†* | 13 | 3 | 29·9 ± 13·9§ |
| 13 | I + BF | + | 15 | 1 | 1 | 0 | 16 | 12 | 4 | 30·6 ± 9·5§ |
| 14 | I + TCPO | + | 9 | 2 | 1 | 0 | 19* | 16 | 2 | 23·6 ± 9·7§ |
| 15 | B[a]P | – | 19 | 15 | 12 | 10 | 7 | 5 | 2 | 42·9 ± 17·7 |
| 16 | B[a]P | + | 14 | 13 | 4 | 4 | 13* | 9 | 3 | 33·5 ± 17·3 |
| 17 | toluene | – | 20 | 18 | 16 | 7 | 0 | – | – | — |
| 18 | toluene | + | 19 | 18 | 15 | 5 | 0 | – | – | — |

* Histology unavailable for one animal.

† One tumour was a spindle-cell sarcoma.

‡ The initiating dose of (I) and B[a]P was 400 μg/mouse; in Group 13, 7,8-benzoflavone (BF) (1,200 μg/mouse) was applied simultaneously; mice in Group 14 received 1,1,1-trichloropropene oxide (TCPO) (10 μl of a 10% v/v solution) at 30 min before and again immediately before the initiating dose of (I).

§ *P*12 *vs* 13 = 0·62; *P*12 *vs* 14 = 0·17, estimated by the method of Mantel (1966)—see Materials and Methods.

FIG. 4.—Induction of dorsal skin tumours after injection of the ketone (I) (3 mg/mouse) followed by promotion with croton oil 8 days (○—○), or 6 months (●—●) later.

tected during the 18th week after intragastric treatment of rats with 30 mg of the carcinogen (I), but the single spontaneous tumour did not appear in the untreated group until the 42nd week. Differences in tumour incidence between the two groups was significant at 20 weeks and highly significant at 50 weeks (Table V). By contrast, benign fibroadenomas occurred later, and to a similar extent in both groups.

### DISCUSSION

In both the repeated-application and initiation-promotion experiments 15,16-dihydro-11-methylcyclopenta[a] phenanthren-17-one (I) is comparable with benzo[a]pyrene as a carcinogen, despite the former having only 3 fused aromatic rings. Practically all known carcinogenic polycyclic hydrocarbons have 4 or more fused aromatic rings, 3 of which form a phenanthrene ring system. The ketone (I), in common with a number of other phenanthrene-derived carcinogens (Jerina *et al.*, 1978) is metabolically activated by conversion to its bay-region, 3,4-dihydro-3,4-dihydroxy-1,2-dihydro-1,2-epoxide (Coombs *et al.*, 1979). It therefore seems probable that (I) represents the smallest conjugated system that can be activated by this mechanism to display strong carcinogenic activity. No evidence for carcinogenicity was found for its 1,2,3,4-tetrahydro derivative (IV), in keeping with the proposed activation mechanism, for this compound lacks the necessary double bonds for metabolic formation of a bay-region diol-epoxide.

At the lowest dose (5 μg twice weekly) co-administration of the aryl-hydrocarbon-

TABLE III.—*Skin tumours appearing on promoted skin following injection of (I) into mice*

| Group | Compound | Injected dose (mg) | Promotion (twice weekly croton oil) | No. of tumour-free survivors at (months) 6 | 12 | 18 | 24 | No. of mice with dorsal tumours | No. of squamous papillomas | No. of squamous carcinomas | Mean latent period ± s.d. (weeks) |
|---|---|---|---|---|---|---|---|---|---|---|---|
| 19 | I | 3 | none | 20 | 16 | 5 | 0 | 0 | – | – | — |
| 20 | I | 3 | 8 days later | 18 | 13 | 0 | 0 | 13 | 9 | 4 | 33 ± 11·3 |
| 21 | I | 3 | 6 months later | 18 | 11 | 7 | 2 | 9 | 8 | 1 | 49* ± 7·0 |
| 22 | I | 0·3 | 8 days later | 19 | 18 | 10 | 5 | 0 | – | – | — |
| 23 | I | 0·3 | 6 months later | 19 | 19 | 14 | 11 | 0 | – | – | — |
| 24 | V | 3 | none | 19 | 19 | 14 | 8 | 0 | – | – | — |
| 25 | V | 3 | 8 days later | 19 | 18 | 16 | 12 | 0 | – | – | — |
| 26 | olive oil | | none | 20 | 19 | 18 | 14 | 0 | – | – | — |

* 24 weeks from beginning of promotion.

TABLE IV.—*Tumours other than those appearing on promoted skin, after injection of (I) into mice*

| Group | Eyelid | Ear | Head | Ventral surface | Mice with lung adenomas‡ |
|---|---|---|---|---|---|
| 19 | 4 (2 pa.)* | — | 2 sq. pa. | 2† (1 mammary ca.) | 4 |
| 20 | 4 sq. pa.<br>2 anaplastic sq. ca.<br>1 sebaceous adenoma | 1 sq. ca. | — | 1 sq. pa. | 7 |
| 21 | 1 sq. pa.<br>1 sq. ca. | 1 sq. ca. | — | 1 sq. pa. | 8 |

* Histology not available for 2 tumours.
† Histology not available for 1 tumour.
sq. pa. = squamous papilloma.
sq. ca. = squamous carcinoma.
‡ No lung adenomas among animals in the olive-oil control (Group 26).

TABLE V.—*Mammary tumours induced in rats after intragastric instillation of (I) (30 mg/rat)*

| | No. of rats at time of treatment | No. of rats with tumour at 20 weeks | No. of rats alive at 20 weeks | No. of rats with tumour at 30 weeks | No. of rats alive at 30 weeks | No. of rats with tumour at 50 weeks | No. of rats alive at 50 weeks | No. of rats with tumour at 75 weeks | No. of rats alive at 75 weeks |
|---|---|---|---|---|---|---|---|---|---|
| Adenocarcinomas | | | | | | | | | |
| In rats after I | 27 | 2 | 26 | 4 | 26 | 6 | 20 | – | — |
| In untreated rats | 96 | 0 | 94 | 0 | 92 | 1 | 85 | – | — |
| *P** | | 0·455 | | 0·0019 | | 0·0001 | | – | — |
| Fibroadenomas | | | | | | | | | |
| In rats after I | 27 | 0 | 26 | 0 | 26 | 0 | 20 | 2 | 13 |
| In untreated rats | 96 | 0 | 94 | 0 | 92 | 3 | 85 | 7 | 70 |
| *P** | | – | — | – | — | 0·5270 | | 0·2845 | |

* Exact value of *P* calculated by method of Yates (Fisher, 1954).

hydroxylase inhibitor, 7,8-benzoflavone (15 μg) reduced the carcinogenicity of (I), in agreement with previous observations (Coombs *et al.*, 1975). Using the dose-response curve shown in Fig. 2, BF apparently causes about 40% inhibition of tumour production under these conditions. It was therefore surprising that inhibition was lacking when BF (1,200 μg) was given together with the initiating dose of I (400 μg) in the two-stage experiment. The reason for this difference is not clear. Several workers have shown that BF inhibits hydrocarbon-induced aryl hydrocarbon hydroxylase more than the constitutive enzyme (Grundin *et al.*, 1973; Hill & Shih, 1975; Wiebel *et al.*, 1971). It seems possible that promotion experiments using a relatively large single dose, as described here, might differ from experiments in which a small dose is administered repeatedly, in that the majority of the dose would be activated by the constitutive enzyme in the first situation, but not in the second.

Shortening of the mean latent period was obtained when the epoxide-hydratase inhibitor TCPO was given topically together with the initiation dose of (I). Enhancement of tumour production with TCPO has also been reported for 3-methylcholanthrene (Berry *et al.*, 1977; Burki *et al.*, 1974) and benzo[a]pyrene (Berry *et al.*, 1977). With the latter, the inhibitor *in vitro* prevents hydration of the initially formed 7,8-oxide to the 7,8-diol (Selkirk *et al.*, 1974) but the ultimate carcinogen, the 7,8-dihydroxy-9,10-epoxide, is apparently not a substrate for this enzyme (Wood *et al.*, 1976). The carcinogen (I) is activated in a manner analogous to that of benzo[a]pyrene, so it seems probable that the mechanism by which TCPO causes enhancement is the same in both cases. Possibly, by inhibiting cytoplasmic epoxide hydratase, TCPO allows the initially-formed non-bay region oxide to escape to a site where it can be more advantageously further converted into the ultimate carcinogen.

In the experiments so far described involving topical application of (I), tumour formation is confined to the treated skin. The injection (mouse) and feeding (rat) experiments demonstrate that this carcinogen is also active systemically, and in more than one animal species. Previously, injection of (I) (8 and 50 mg) into mice led to ventral skin tumours as well as sarcomas at the site of injection (Coombs & Croft, 1969). Tumours at several sites, including the ventral surfaces, were seen in the present experiment (Table IV). Injection of 3 mg per mouse, but not one tenth of this dose, was sufficient to initiate the dorsal skin (Fig. 4 and Table III) so that subsequent promotion yielded a skin-tumour incidence of 65% and a mean latent period of 33 weeks. After injection initiation persisted, for when promotion was started 6 months later skin tumours occurred in 50% of the mice, but with a shorter mean latent period of 24 weeks (calculated from the beginning of promotion). No tumours were induced with the parent unsubstituted ketone (V) when injection of this compound (3 mg) was followed by topical treatment with croton oil. This agrees with the failure to induce tumours in mice by injection (50 mg) or by skin painting (50 $\mu$g twice weekly) (Coombs & Croft, 1969) or in the two-stage system with an "initiating" dose of 400 $\mu$g (Coombs & Bhatt, 1978).

In its ability to induce mammary carcinomas after a single intragastric instillation of 30 mg, compound (I) resembles other known potent carcinogens such as 3-methylcholanthrene (Shay *et al.*, 1949) and 7,12-dimethylbenz[a]anthracene (Huggins, 1961). However, it is less potent than either of these, both in this regard and also as judged by their relative mean latent periods for induction of skin tumours in mice (Coombs & Croft, 1969). The suggestion that the carcinogenicity of aromatic hydrocarbons increases as their structure approaches that of the steroids (Yang *et al.*, 1961) is therefore not substantiated. The ketone (I) not only possesses the same carbon-ring system as the steroids, but also bears an oxygen atom at C-17, a position which is oxygenated in virtually all natural $C_{18}$ and $C_{19}$ steroids. This carcinogen, with potency similar to that of benzo[a]pyrene, is best considered as a simple member of the large group of polycyclic hydrocarbon carcinogens whose structures are based on phenanthrene.

We thank Dr L. Pang for confirming the pathology of the mouse tumours, Miss J. Macdonald for valuable assistance, and Mr G. D. Everitt for providing computer facilities.

### REFERENCES

BERRY, D. L., SLAGA, T. J., VIAJE, A. & 4 others (1977) Effect of trichloropropene oxide on the ability of polyaromatic hydrocarbons and their "K-region" oxides to initiate skin tumours in mice and to bind to DNA *in vitro*. *J. Natl Cancer Inst.*, **58**, 1051.

BRYAN, W. R. & SHIMKIN, M. B. (1941) Quantitative analysis of dose-response data obtained with carcinogenic hydrocarbons. *J. Natl Cancer Inst.*, **1**, 807.

BÜRKI, K., WHEELER, J. E., AKAMATSU, Y., CANDELAS, G. & BRESNICK, E. (1974) Early differential effects of 3-methyl-cholanthrene and its "K-region" epoxide on mouse skin. Possible implication in the two stage mechanism of tumorigenesis. *J. Natl Cancer Inst.*, **53**, 967.

COOMBS, M. M. (1966) Potentially carcinogenic cyclopenta[a]phenanthrenes. I. A new synthesis of 15,16-dihydro-17-oxocyclopenta[a]phenanthrene and the phenanthrene analogue of 18-noroestrone methyl ether. *J. Chem. Soc.* (C), 955.

COOMBS, M. M. & BHATT, T. S. (1973) Potentially carcinogenic cyclopenta[a]phenanthrenes. VI. 1,2, 3,4-tetrahydro-17-ketones. *J. Chem. Soc.* (*Perkin* I), 1251.

COOMBS, M. M. & BHATT, T. S. (1978) Lack of initiating activity in mutagens which are not carcinogenic. *Br. J. Cancer*, **38**, 148.

COOMBS, M. M., BHATT, T. S. & CROFT, C. J. (1973) Correlation between carcinogenicity and chemical structure in cyclopenta[a]phenanthrenes. *Cancer Res.*, **33**, 832.

COOMBS, M. M., BHATT, T. S. & VOSE, D. W. (1975) The relationship between metabolism, DNA binding, and carcinogenicity of 15,16-dihydro-11-methylcyclopenta[a]phenanthren-17-one in the presence of a microsomal enzyme inhibitor. *Cancer Res.*, **35**, 305.

COOMBS, M. M. & CROFT, C. J. (1969) Carcinogenic cyclopenta[a]phenanthrenes. *Prog. Exp. Tumor Res.*, **11**, 69.

COOMBS, M. M., KISSONERGHIS, A.-M., ALLEN, J. A. & VOSE, C. W. (1979) Identification of the proximate and ultimate forms of the carcinogen 15,16-dihydro-1-methylcyclopenta[a] phenanthren-17 one. *Cancer Res.*, **39**, 4160.

FISHER, R. A. (1954) *Statistical Methods for Research Workers*, 12th ed. Edinburgh: Oliver and Boyd. p. 96.

GRUNDIN, R., JACKOBSSON, S. & CINTI, D. L. (1973) Induction of microsomal aryl hydrocarbon (3,4-benzo[a]pyrene) hydroxylase and cytochrome P-$450_K$ in rat kidney cortex. *Arch. Biochem. Biophys.*, **158**, 544.

HILL, D. L. & SHIH, T.-W. (1975) Inhibition of benzo[a]pyrene metabolism catalysed by mouse and hamster lung microsomes. *Cancer Res.*, **35**, 2717.

HUGGINS, C. (1961) Rapid induction of mammary cancers and their suppression. *Science*, **133**, 1366.

JERINA, D. M., YAGI, H., LEHR, R. E. & 7 others (1978) The bay-region theory of carcinogenesis by polycyclic hydrocarbons. In *Polycyclic Hydrocarbons and Cancer*. Vol. 1, Ed. Gelboin and Ts'O. London: Academic Press. p. 173.

MANTEL, N. (1966) Evaluation of survival data and two new rank order statistics arising in its consideration. *Cancer Chemother. Rep.*, **50**, 163.

SELKIRK, J. K., CROY, R. G., ROLLER, P. P. & GELBOIN, H. V. (1974) High pressure liquid chromatographic analysis of benzo[a]pyrene metabolism and covalent binding and the mechanism of action of 7,8,benzoflavone and 1,2-epoxy-3,3,3-trichloropropane. *Cancer Res.*, **34**, 3474.

SHAY, H., AEGERTER, E. A., GRUENSTEIN, M. & KOMAROV, S. A. (1949) Development of adenocarcinoma of the breast in the Wistar rat following gastric instillation of methylcholanthrene. *J. Natl Cancer Inst.*, **10**, 255.

WIEBEL, F. J., LEUTZ, J. C., DIAMOND, L. & GELBOIN, H. V. (1971) Arylhydrocarbon (benzo-[a]pyrene) hydroxylase in microsomes from rat tissues: Differential inhibition and stimulation by benzoflavones and organic solvents. *Arch. Biochem. Biophys.*, **144**, 78.

WOOD, A. W., WISLOCKI, P. G., CHANG, R. L. & 6 others (1976) Mutagenicity and cytotoxicity of benzo[a]pyrene benzo-ring epoxides. *Cancer Res.*, **36**, 3358.

YANG, N. C., CASTRO, A. J., LEWIS, M. & WONG, T. W. (1961) Polynuclear hydrocarbons, steroids, and carcinogenesis. *Science*, **134**, 386.

YOUNG, S. & HALLOWES, R. C. (1973) Tumours of the mammary gland. In *Pathology of Tumours in Laboratory Animals*, Vol. 1. *Tumours of the Rat*, Part 1. Ed. Turusov. Lyon: IARC Scientific Pub. p. 31.